# Involvement of the Wnt/β‐catenin signalling pathway in heterotopic ossification and ossification‐related diseases

**DOI:** 10.1111/jcmm.70113

**Published:** 2024-09-25

**Authors:** Yike Zhao, Fangzhou Liu, Yiran Pei, Fengyu Lian, Hui Lin

**Affiliations:** ^1^ Department of Pathophysiology, School of Basic Medical Sciences, Jiangxi Medical College Nanchang University Nanchang Jiangxi China; ^2^ Queen Mary school, Jiangxi Medical College Nanchang University Nanchang Jiangxi China

**Keywords:** heterotopic ossification, molecular mechanism, therapeutic measures, Wnt/β‐catenin signalling

## Abstract

Heterotopic ossification (HO) is a pathological condition characterized by the formation of bone within soft tissues. The development of HO is a result of abnormal activation of the bone formation programs, where multiple signalling pathways, including Wnt/β‐catenin, BMP and hedgehog signalling, are involved. The Wnt/β‐catenin signalling pathway, a conserved pathway essential for various fundamental activities, has been found to play a significant role in pathological bone formation processes. It regulates angiogenesis, chondrocyte hypertrophy and osteoblast differentiation during the development of HO. More importantly, the crosstalk between Wnt signalling and other factors including BMP, Hedgehog signalling, YAP may contribute in a HO‐favourable manner. Moreover, several miRNAs may also be involved in HO formation via the regulation of Wnt signalling. This review aims to summarize the role of Wnt/β‐catenin signalling in the pathogenesis of HO, its interactions with related molecules, and potential preventive and therapeutic measures targeting Wnt/β‐catenin signalling.

## 1 INTRODUCTION

Heterotopic ossification (HO) is the abnormal formation of bone within soft tissue. It was initially observed in soldiers who suffered severe trauma during World War I. Apart from trauma, neurogenic injuries and certain genetic factors can also contribute to the development of HO.^1^, ^2^, ^3^, ^4^ HO can be categorized into two types: acquired HO (aHO), which is the most common type, and hereditary HO, including fibrodysplasia ossificans progressiva (FOP), progressive osseous heteroplasia (POH) and Albrigh hereditary osteodystrophy (AHO).^5^, ^6^ In the early stages of HO, symptoms are nonspecific and may include signs of inflammation, such as fever, swelling, erythma and pain, accompanied by reduced joint mobility. As the disease progresses, joint mobility further decreases, significantly impacting the patient's quality of life. While approximately 80% of patients with HO do not develop serious complications, the remaining 20% may experience joint ankylosis, severe loss of mobility and other complications.^4^, ^5^

**FIGURE 1:**
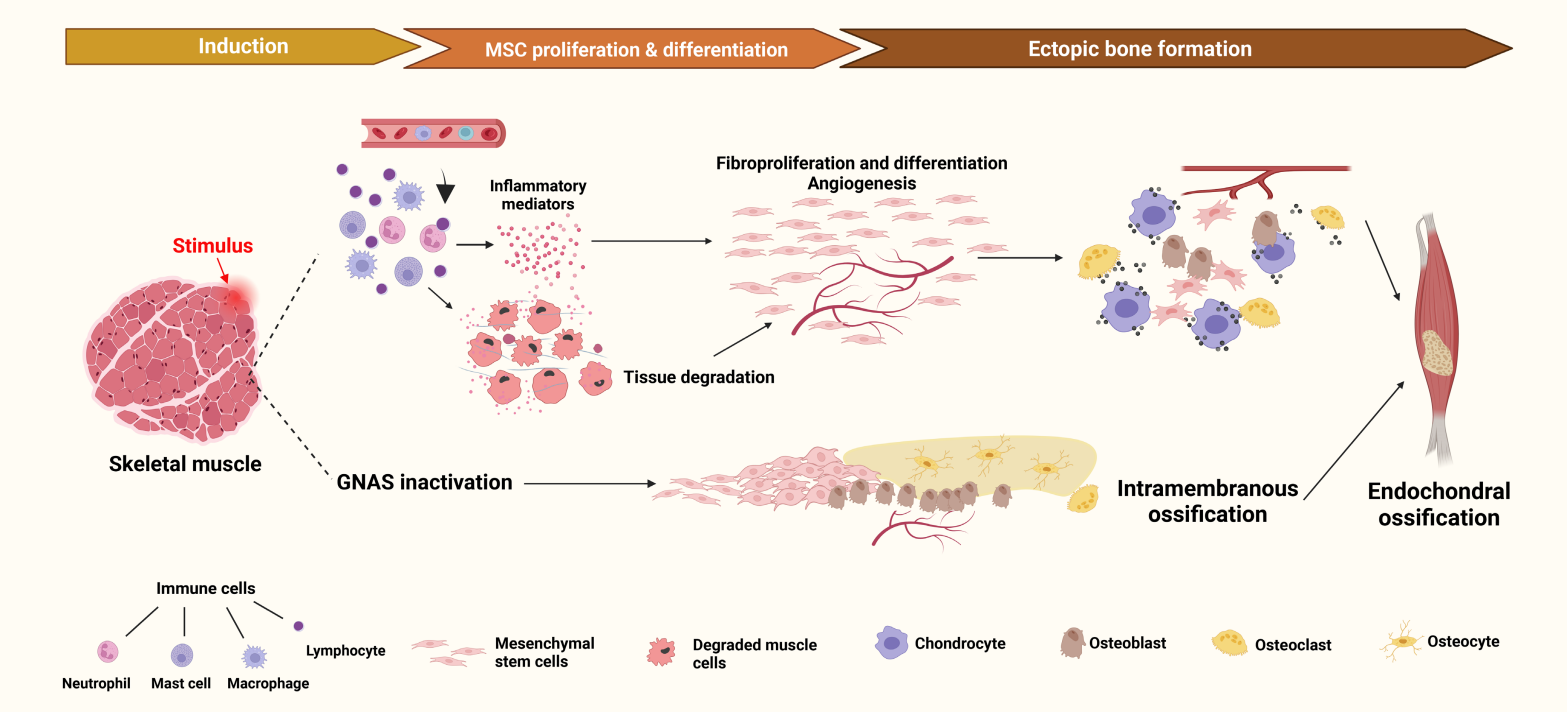
Overview of HO development. The figure illustrates the development of HO through endochondral or intramembranous pathways with tHO and POH as examples. Damage caused by external stimuli induces tissue inflammation, accompanied by the recruitment of various inflammatory cells, including neutrophils, monocytes and mast cells. The release of inflammatory mediators not only helps clear damaged tissue but also promotes the proliferation and differentiation of fibroblasts, thus initiating the repair process where angiogenesis begins and fibroblasts accumulate. Furthermore, local factors produced by degenerated tissue stimulate the proliferation of MSCs and result in their chondrogenic differentiation. GNAS inactivation leads to POH through intramembranous ossification, where MSCs directly differentiate into osteoblasts. Due to the reactivation of the bone formation program, both processes involve a variety of cells, including chondrocytes, osteoblasts, osteoclasts and osteoclasts. (Created with BioRender.com).

The development of HO involves the reactivation of bone formation programs, the proliferation and differentiation of osteogenic and chondrogenic progenitor cells, and eventually bone maturation. Osteogenic induction factors and a suitable environment are indispensable in this process.^7^ Inductive factors recruit mesenchymal stem cells (MSCs) to proliferate and differentiate, forming ectopic bone through either intramembranous or endochondral ossification pathways.^8^ Inflammation plays a significant role in the induction of HO. Acquired HO (aHO) is assumed to go through this process followed by endochondral ossification.^9^, ^10^ FOP is highly susceptible to HO development due to genetic factors, where inflammation can accelerate this process.^11^, ^12^ Inflammatory mediators lead mesenchymal stem cells (MSCs) within soft tissue to first differentiate into chondrocytes, forming a cartilage model that serves as a scaffold for subsequent osteogenic differentiation. Accompanied by chondrocyte hypertrophy and surrounding cartilage matrix calcification, blood vessels extending from the periosteum transport osteoblasts, osteoclasts and mesenchymal stem cells to the site of bone tissue generation and maturation.^13^ Nevertheless, inflammation is rarely observed in the intramembranous ossification process, that is, POH and AHO, whose exact mechanism of development remains unclear. In this context, MSCs differentiate directly into osteoblasts and form mature ectopic bone under the regulation of a series of signals.^14^, ^15^ (Figure 1).

**FIGURE 2:**
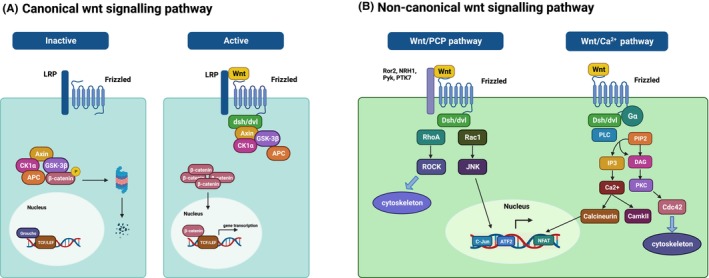
Canonical and noncanonical Wnt signalling pathways. Wnt receptors consist of the Frizzled (Fzd) family receptors and the low‐density lipoprotein receptor‐related protein (LRP) family (LRP5 or LRP6) and are located on the cell membrane. (A) Normally, in the absence of Wnt ligand stimulation, the effector mediator was caught by the tetramer consisting of APC, GSK3β, Axin and CK1a and is continuously phosphorylated by active GSK3β, leading to degradation of β‐catenin. In the nucleus, the transcription factor TCF/LEF binds to the repressor Groucho to repress Wnt target gene expression. Once Wnt binds to the coreceptor, the cytoplasmic mediator dishevelled (dsh/dvl) is activated and leads to GSK3β inactivation. In this way, β‐catenin is stabilized and translocated to the nucleus. In fact, β‐catenin itself does not direct gene transcription but binds to the transcription factor TCF/LEF, where β‐catenin replaces the negative regulator Groucho, thereby directing the transcription of Wnt target genes. The noncanonical pathways are more complex. (B) In the noncanonical planar cell polarity pathway, cascading events activate Rho‐associated protein kinase (ROCK) and target genes, thereby remodelling the actin backbone. In the noncanonical Wnt/calcium pathway, activated Fzd acts on dsh/dvl and G proteins to induce PLC and PDE pathways, thus regulating the release of calcium from the endoplasmic reticulum. (Created with BioRender.com).

This process involves multiple signalling pathways, including the TGF‐β family, hedgehog (hh) and Wnt signalling pathways.^16^, ^17^, ^18^, ^19^ The Wnt signalling pathway is a conserved pathway that is involved not only in physiological developmental processes, such as specification of embryonic axes, self‐renewal of adult cells and maintenance of reproductive capacity but also in pathological processes, such as cancers, degenerative diseases and bone diseases.^20^ Currently, 3 Wnt pathways have been identified: the canonical Wnt signalling pathway (also known as the Wnt/β‐catenin pathway), the noncanonical planar cell polarity pathway (or Wnt/PCP pathway) and the noncanonical Wnt/calcium pathway (or Wnt/Ca2+ pathway)^6^ (Figure 2). Physiologically, Wnt signalling regulates the activity of osteoblasts and chondrocytes to modify bone formation while also impacting various aspects of bone mass and fragility. Similarly, the role of Wnt in pathological bone formation has been reported, primarily focusing on chondrogenesis and osteogenesis. However, compared to organized cellular events in normal bone formation, these events in HO are disorganized due to abnormal pathological stimuli and asynchronous cellular activities.^21^ Furthermore, the involvement of noncanonical Wnt signalling in ossification has been claimed, with more complex mechanisms identified.^6^ This review will predominantly focus on the significant role played by the canonical Wnt signalling pathway in different stages of HO development, aiming to complement the mechanism of HO and provide a foundation for the development of therapeutic pathways targeting Wnt/β‐catenin signalling.

## 2 CONTRIBUTION OF WNT/Β‐CATENIN SIGNALLING TO HO

**FIGURE 3:**
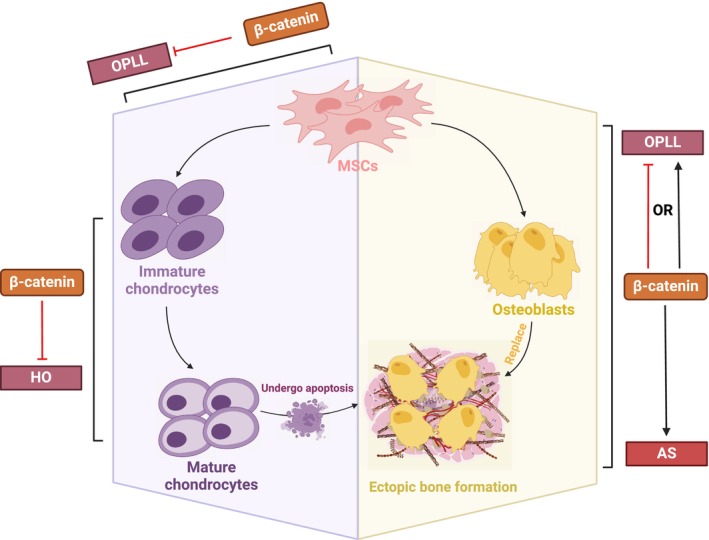
Effects of Wnt/β‐catenin signalling on stages of HO. The involvement of Wnt signalling was mainly seen in endochondral ossification as shown in the figure. Wnt signalling promotes chondrocyte maturation in the background of HO. Wnt signalling enhances the osteogenesis in AS while controversial effects have been seen in OPLL in terms of osteoblast differentiation. It also inhibits the differentiation of MSCs to chondrocytes which hinders the development of ossification. (Created with BioRender.com).

Wnt signalling in the early repair and osteogenic stages of HO has been shown to contributes to the development of HO by promoting angiogenesis^22^ and a series of events involved in osteogenesis, including the commitment of MSCs to osteoblasts, differentiation and maturation of osteoblasts.^23^ Additionally, studies have shown complex effects of Wnt signalling on regulating the chondrocyte differentiation (Figure 3).

### 2.1 Wnt/β‐catenin signalling in the repair process

Although the role of Wnt signalling in the regulation of angiogenesis has been previously elucidated by Sébastien et al.,^24^ a recent study has revealed the role of canonical Wnt signalling in regulating angiogenesis in HO. Previous studies have shown that bone is a highly vascularized tissue that provides the necessary nutrients and oxygen for its generative processes and is essential for both physiological and pathological osteogenesis. Additionally, the presence of type‐H vessels in HO bone tissue and its subsequent coupling to bone tissue have been reported to be of great significance.^25^ Guo et al. demonstrated that the posttraumatic YAP/β‐catenin axis mediated changes in angiogenesis and osteogenic activity in endothelial cells (ECs) and tendon‐derived stem cells (TDSCs), respectively. Treatment with verteporfin, a YAP inhibitor, significantly downregulated the expression of YAP and β‐catenin in human umbilical vein endothelial cells (HUVECs), whose changes were positively correlated, indicating a regulatory relationship between YAP and β‐catenin.^22^ The results of both in vivo and in vitro experiments showed decrease in the proliferative and migratory activities of HUVECs, along with downregulation of proangiogenic factors, including VEGF, CD31 and EMCN, which inhibited angiogenesis and weakened the ensuing osteogenic activities. Additionally, the results demonstrated the inhibitory effect of verteporfin, mediated by YAP/β‐catenin, on the osteogenic differentiation of TDSCs.^22^

### 2.2 Wnt/β‐catenin signalling in chondrogenic and osteogenic differentiation

#### 2.2.1 Wnt/β‐catenin signalling regulates the differentiation of MSCs

MSCs in soft tissues have the ability to differentiate into osteoblasts, chondrocytes and adipocytes, representing three distinct cell lineages.^26^ Previous studies have demonstrated the key role of Wnt/β‐catenin signalling pathway in regulating this process. It promotes osteoblast differentiation by upregulating the expression of osteoblast‐specific genes, such as Runx2, Osterix (osx) and ALP.^27^, ^28^, ^29^ Additionally, it inhibits chondrocyte and adipocyte differentiation by suppressing the expression of Sox9, CEBPα and PPARγ.^27^, ^29^, ^30^

A study on ankylosing spondylitis (AS) found that miR‐124 was abnormally elevated in fibroblasts from ligament tissue of AS patients, which enhanced the expression of β‐catenin by inhibiting the function of GSK‐3β, and subsequently upregulated the expression of osx and runx2. Then the activity of the osteogenic factors was suppressed by inhibiting miR‐124 and β‐catenin,^27^ suggesting that miR‐124, a dysregulated factor, promotes the process of osteogenic differentiation of fibroblasts through activation of the Wnt pathway and contributes to the generation of AS. Additionally, the mechanical load, which has been reported as the inductive factor both in OPLL and AS, has also been reported to promote osteogenic activities via the activity of β‐catenin.^31^, ^32^ In contrast, however, a study on OPLL demonstrated that Wnt signalling inhibited the process of osteogenic differentiation in patient‐derived ligament fibroblasts. Osterix was abnormally upregulated in patient‐derived ligament fibroblasts from OPLL and dexamethasone‐induced ossification of normal spinal ligament cells, respectively, leading to increased osteogenic activity. More detailed, it inhibited Wnt activity by inducing the production of dkk‐1 and sost production to inhibit Wnt signalling, whereas elevated β‐catenin expression suppressed ALP, OCN and COL‐1 expression and inhibited the process of ossification.^33^ In comparison, different expression of β‐catenin can be observed in different pathological osteogenic background and it exerted opposite impact on regulating osteogenic factors. Another study revealed the role of Wnt signalling in inhibiting ligament cell differentiation into the chondrogenic lineage in the development of OPLL. OPLL involves the endochondral ossification of the injured posterior spinal ligament, and the downregulation of RSPO2 gene expression has been identified as a susceptibility factor in OPLL patients.^34^ RSPO2 acts as a negative regulator in the chondrogenic differentiation of MSCs, and its downregulation results in increased chondrogenic differentiation. It functions as an agonist of the Wnt signalling pathway and exhibits a high affinity for Wnt receptors, including G protein‐coupled receptors, LRP5 and LRP6.^35^ By promoting the expression of Wnt3a, RSPO2 could inhibit aberrant chondrogenic differentiation of ligament MSCs in the pathogenesis of OPLL, accompanied by decrease of Col2a1, Acan and Sox9. In this context, the expression of canonical Wnt signalling blocks the aberrant differentiation of MSCs into chondrocytes, enabling MSCs to differentiate into ligament cells for tissue repair, while downregulated RSPO2 leads to a lack of Wnt signalling, resulting in OPLL.^34^

#### 2.2.2 Wnt/β‐catenin signalling is responsible for final maturation, including hypertrophy and mineralization of chondrocytes

Although the Wnt/β‐catenin pathway inhibits the chondrocyte fate of MSCs, it promotes the terminal maturation of chondrocytes.^36^ In vitro studies using chicken chondrocytes have reported that the Wnt/β‐catenin pathway increases the expression of MMP‐13, MMP‐2, MMP‐9 and osteopontin, indicating chondrocyte hypertrophy and mineralization of the surrounding matrix.^37^ Notably, translocation of β‐catenin to the nucleus only occurred in hypertrophic chondrocytes, while β‐catenin in immature chondrocytes remained in the cytoplasm, highlighting the significance of Wnt/β‐catenin signalling in the final maturation of chondrocytes. This event involving Wnt plays a crucial role in facilitating the subsequent replacement of bone tissue and initiating endochondral ossification.^37^ In another study, Kitagaki et al. used chick chondrocytes infected with retroviral vectors encoding constitutively active (CA) or dominant‐negative (DN) forms of LEF for the purpose of activating or silencing Wnt/β‐catenin signalling, respectively, and then injected chick chondrocytes intramuscularly into nude mice to test for the formation of ectopic bone.^20^ The results demonstrated that Wnt signalling activation promotes the formation of ectopic bone in mice, whereas inhibition of Wnt signalling hinders ectopic bone formation. Analysis of Wnt proteins in the cartilage structures of chick and mouse embryos revealed that overexpression of Wnt8 accelerated chondrocyte maturation. Therefore, Wnt8 may be a key factor in promoting chondrocyte maturation and determining ectopic bone formation.^20^ Mechanistically, studies have shown that Wnt signalling interacts with factors such as Runx2 and Sox9 in this process.^30^

Combined with the previous finding that Wnt signalling impeded the differentiation of MSCs to chondrocyte lineage, it can be concluded that the expression of wnt/β‐catenin signalling may have promotive or inhibitory effect on pathological endochondral ossification depending on the phases of chondrogenic differentiation.

## 3 THE CROSSTALK BETWEEN WNT/Β‐CATENIN SIGNALLING AND OTHER FACTORS INVOLVED IN HO DEVELOPMENT

**FIGURE 4:**
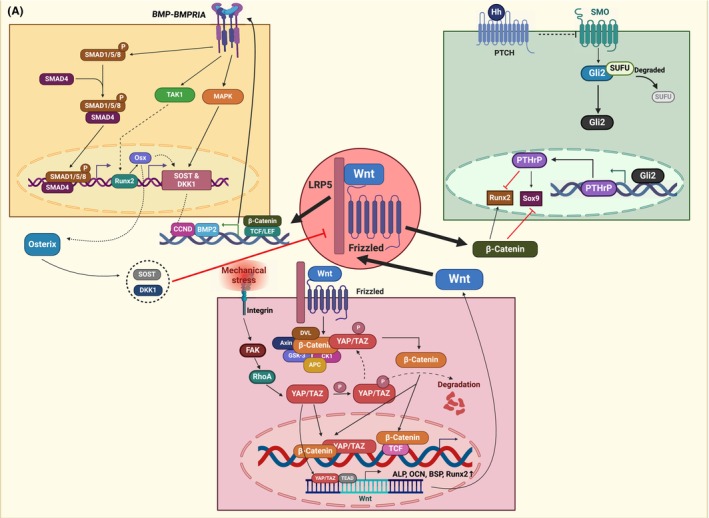
Important osteogenic factors regulating Wnt/β‐catenin signalling in HO. This figure illustrates the regulation of Wnt signalling, including BMP, hh and YAP. BMP interacts with Wnt signalling through the smad pathway, and factors, including Runx2 and osx, are involved in regulating a series of events. HH signalling and Wnt exert opposite effects on chondrogenesis by targeting Runx2 and Sox9. Phosphorylated YAP is involved in the degradation of β‐catenin by incorporation in the tetramer complex, while active YAP assists in the stabilization of β‐catenin and the expression of Wnt ligands.

**FIGURE 5:**
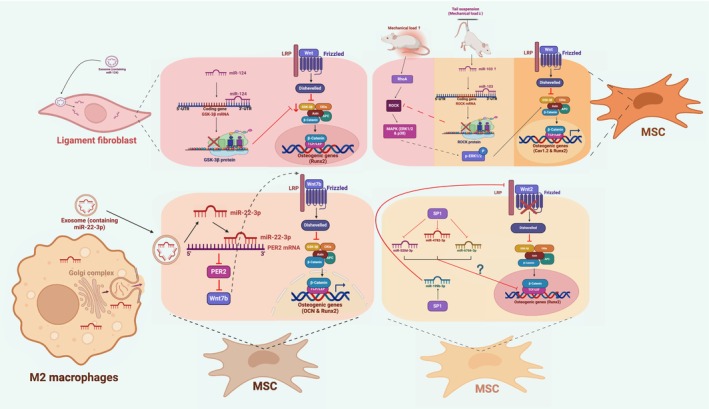
miRNAs regulating Wnt/β‐catenin signalling in HO. Various miRNAs upstream activate or inhibit Wnt signalling in different cells. miR‐124: In ligament fibroblasts (similar to MSCs), miR‐124 incorporation could bind to the 3'‐UTR of GSK‐3β mRNA to block GSK‐3β protein synthesis, which could reduce the trapping ability of the AXIN/APC/CK1/GSK‐3β complex to promote the nuclear translocation of β‐catenin, helping gene (Runx2) transcription. miR‐103: Pathologically, increased mechanical load could activate RhoA and further ROCK to induce the phosphorylation of MAPK pathways, including p38 and ERK1/2, therefore activating the Dvl/Axin/GSK‐3β/CK1/APC complex to release β‐catenin to bind with TCF/LEF, helping the transcription of osteogenic genes, including Cav1.2 and Runx2. However, tail suspension could reduce the mechanical burden to increase miR‐103 expression to bind with the 3'‐UTR of ROCK to block ROCK activity. As a result, Wnt signalling was reduced to alleviate osteogenesis. miR‐520d‐3p, miR‐4782‐3p, miR‐6766‐3p and miR‐199b‐3p: SP1 expression could reduce the amount of miR‐520d‐3P, miR‐4782‐3p and miR‐6766‐3p, which may promote osteogenic gene expression. In addition, SP1 could also elevate miR‐199b‐3p to inhibit Wnt2, which potentiates Wnt signalling for osteogenesis. miR‐22‐3p: M2 macrophage‐derived exosomes containing miR‐22‐3p could bind with PER mRNA that could block PER protein synthesis, therefore disinhibiting Wnt7b expression to strongly activate Wnt signalling to promote osteogenic gene expression, including Runx2 and OCN. (Created with BioRender.com).

Many dysregulated factors can contribute to HO via Wnt/β‐catenin signalling, which involves some signalling pathways reported to be involved in HO, including BMP and Hedgehog signalling. Additionally, aberrant expression of YAP and some miRNAs induced by other stimuli also exerts impacts on Wnt signalling.(Figures 4,5).

### 3.1 BMP/TGF‐β

BMPs are highly potent growth factors that play a crucial role in promoting bone formation and are considered to be the primary signals in HO.^38^ In a case of adrenal myelolipoma (ML) with HO, the abnormal expression of BMP2 and Wnt/β‐catenin signalling were suspected to be responsible for the induction of ossification. The Wnt pathway has been observed to stimulate the expression of BMP2 and its target genes, suggesting that Wnt acts as an upstream inducer of BMP2 expression in osteoblasts. The collaboration between Wnt/β‐catenin and BMP2 directs the differentiation of bone marrow MSCs toward osteoblasts, which are the basis for HO formation.^39^ Consistently, it has been reported that the expression of Wnt signalling can induce the expression of BMP2 in normal skeletal development.^40^ Moreover, BMP9 and Wnt/β‐catenin signalling have been found to jointly contribute to HO in colon cancer.^16^ Both BMP and TGF‐β serve as chondrogenic cytokines that induce chondrogenesis and were found to appear in ossification site,^41^ while the upregulation of chondrogenic differentiation markers induced by BMP‐2 and TGF‐β1 can be inhibited by Wnt3a signalling enhanced by RSPO2.^34^, ^41^ These studies have highlighted the bidirectional regulatory relationship between BMP and Wnt signalling during ossification. However, our current understanding of the interaction between these pathways under the background of HO remains limited, and further research is needed for a comprehensive elucidation.^6^

### 3.2 Hedgehog (hh) signalling pathway

Kingston et al. established a mouse embryo model with ptch^−/−^ mutations specific to chondrocytes and partial osteoblasts and found that overactivated hh signalling led to ectopic bone formation in the articular perichondrium. However, bone formation in ptch^−/−^, β‐catenin^−/−^ double mutant mice, where the expression of β‐catenin was also blocked, was attenuated to the same level as that in β‐catenin^−/−^ single mutant mice.^42^ Mechanistically, it was shown that hh induced the expression of the important osteogenic factors runx2 and osx, which in turn promoted osteoblast maturation. However, subsequent activity needed the involvement of Wnt signalling, suggesting that Wnt/β‐catenin signalling downstream of Ihh mediates osteoblast differentiation. In addition, chondrocyte hypertrophy was significantly attenuated in double mutants compared to β‐catenin^−/−^ single mutants.^42^ This might be attributed to the indirect inhibition of chondrogenic hypertrophy by HH through PTHrp, which acts by repressing the expression of runx2 and sox9. Interestingly, Wnt signalling targets the same genes, runx2 and sox9, but exerts opposite effects to promote the maturation of chondrocytes.^42^ Moreover, the balance of Wnt and hh signalling is critical in POH formation.^43^ Regard et al. elucidated the effects of different expression patterns of hh and Wnt signalling on the outcome of bone formation.^44^ GNAS, as an important factor regulating the differentiation of MSCs, regulates the balance of hh and Wnt signalling, whose aberrant inactivation and overactivation can lead to extreme hh and Wnt signalling, respectively, resulting in bone formation diseases. Excessive levels of Hh signalling together with low levels of Wnt signalling lead to POH.^44^, ^45^ In this case, HH acts as the osteogenic inducer leading to ectopic bone production, but the interaction between the Wnt and hh pathways needs to be further elucidated.

### 3.3 Yes‐associated protein (YAP)

In recent years, there have been reports on the impact of mechanical stress on the development of ossification of OPLL. It signals cells through integrin receptors on the cell membrane, resulting in cytoskeletal remodelling, dephosphorylation of Yes‐associated protein (YAP) and its translocation from the cytoplasm to the nucleus.^46^ Phosphorylated YAP is involved in the formation of complexes that phosphorylate β‐catenin, while dephosphorylated YAP assists in activating β‐catenin, which leads to the upregulation of osteogenic genes, including Runx2, Cola1, Osterix, OCN and ALP.^47^ However, these changes mediated by YAP‐Wnt/β‐catenin have only been observed in OPLL‐derived ligament fibroblasts compared to the control group, suggesting the susceptibility of cells derived from OPLL patients.^32^ Additionally, as mentioned above, YAP is involved in inducing the expression of β‐catenin in ECs and TDSCs, thus promoting angiogenesis and osteogenesis in HO. In addition to benefit the accumulation of β‐catenin, YAP could also bind TEAD to upregulate the expression of the Wnt ligand, which enhances the function of Wnt signalling.^22^

### 3.4 miRNAs

MicroRNAs play a significant role in regulating various biological processes. In recent years, several miRNAs have been found to be involved in physiological osteogenesis and pathological bone diseases through their impact on Wnt‐β‐catenin signalling.^31^, ^48^ Similarly, miRNAs have been found to be responsible for aberrant activation of Wnt signalling in ossification‐related diseases. A study revealed downregulation of miR‐487b in primary cells from patients with OPLL, which may lead to abnormal activation of Wnt/β‐catenin signalling in MSCs and chondrocytes along the ossification front, resulting in increased osteogenic activity.^30^ It is predicted that miR‐487b may affect the function of Wnt pathway coreceptors by targeting and regulating LRP6 gene expression. Additionally, its target genes are involved in the regulators of Wnt signalling, including NRARP, IRS1, PRKCA, EPHA3, POU2F1 and MAP2K4, as well as factors regulating cartilage and osteogenesis, such as BMP, Runx2 and Sox9, among others.^30^ AS is associated with the osteogenic activity of bone marrow mesenchymal stem cells (BMMSCs). A study reported that miR‐22‐3p from macrophage M2 extracellular vesicles promotes osteogenic differentiation of BMMSCs. Abnormally upregulated miR‐22‐3p was found to target and inhibit Periodic Circadian Rhythm Protein 2 (PER2), resulting in reduced expression of Wnt7b, β‐catenin, c‐Myc, Cyclin D1, Runx2 and OCN, which indicated decreased Wnt/β‐catenin signalling and osteogenic activity. Restoration of Wnt7b expression reversed these effects, suggesting that abnormally upregulated miR‐22‐3p promotes the osteogenic process via Wnt signalling in AS.^49^ Similarly, another study reported that upregulated miR‐124 in AS is responsible for the overexpression of β‐catenin. Upregulation of miR‐124 and β‐catenin, along with downregulation of GSK‐3β, was observed in cells derived from AS patients, while blocking miR‐124 expression restored GSK‐3β expression, inhibiting Wnt/β‐catenin signalling and osteoblast differentiation processes. This suggests that miR‐124 promotes the transcriptional activity of β‐catenin by inhibiting GSK‐3β expression, thereby promoting the osteogenic differentiation process of fibroblasts in AS.^27^ Additionally, reducing mechanical loading was found to delay the onset of sacroiliac joint ossification in AS induced by proteoglycan. Mechanosensitive miR‐103 was upregulated in sacroiliac joint tissues upon mouse tail suspension treatment.^31^ Previous studies have shown that mechanical loading activates the ROCK1 kinase and MAPK pathways. MiR‐103 suppressed the expression of ROCK1, p‐Erk, β‐catenin and osteogenesis‐related genes, including BMP2, Runx2 and Bglap, while increasing the expression of dkk1, which suppressed Wnt signalling. These findings suggest that reduced mechanical stress upregulates miR‐103 expression and suppresses Wnt/β‐catenin signalling via ROCK1 and p‐Erk, thereby alleviating the onset of osteogenesis in AS.^31^ A meta‐analysis of OPLL‐associated miRNA molecules demonstrated that dysregulation of the transcription factor SP1 can modulate Wnt signalling through the regulation of multiple miRNAs, leading to OPLL. SP1 downregulates miR‐520d‐3p, miR‐4782‐3p and miR‐6766‐3p and upregulates miR‐199b‐5p to enhance the expression of LEF1 and reduce the inhibitory effect of Wnt2 on Wnt signalling^50^ (Tables 1,2).

**TABLE 1:** miRNAs regulating Wnt signalling in HO.

Factor/molecule	Target	Effect on Wnt signaling	Result	Reference
miR‐487b	Coreceptor	+	Increase osteogenic activity of MSC and chondrocytes	^30^
miR‐22‐3p	PER2↓	+	Increase osteogenic activity	^49^
miR‐124	GSK‐3β↓	+	Promote osteoblast differentiation	^27^
miR‐103	ROCK1, p‐Erk↓	−	Downregulate of osteo‑specific genes (Bmp2, Runx2 and Bglap)	^31^
miR‐520d‐3p	LEF1↑	+	—	^50^
miR‐4782‐3p		+		
miR‐6766‐3p		+		
mir‐199b‐5p	Wnt2↓	+		

**TABLE 2:** Potential Wnt/β‐catenin signalling‐related inhibitors and regulators in HO treatment.

Inhibitor	Disease	Target cells/Tissue	Mechanism	Reference
Verteporfin	tHO	HUVECs and TDSCs	Inhibit YAP and β‐catenin expression to impede angiogenesis and osteogenic differentiation might target expression of LRP5/6, NRARP, IRS1, PRKCA, EPHA3, POU2F1, MAP2K4 and BMP, Runx2 or Sox9 signaling etc. PER2 downregulate Wnt7b Suppressed ROCK1 and MAPK activity, thus decreasing phosphorylation of GSK‐3β activates Wnt inhibitors sost and dkk to impede Wnt signaling interact with BMP and its receptors to activate p38MAPK signaling, which induces expression of sost and dkk1	^22^
miR‐487b‐3p	OLF	Posterior longitudinal ligament cells		^30^
PER2	AS	BMSC		^49^
miR‐103	AS	293T cells		^32^
Osterix	OPLL	OPLL‐derived ligament cells		^33^
Tfr2	HO	Mice osteoblast and muscle		^70^

## 4 PREVENTION AND TREATMENT OF HO BY TARGETING WNT/Β‐CATENIN SIGNALLING

Therapies for HO can be classified into prophylactic strategies and treatments for established HO. To prevent the development of HO, nonsteroidal anti‐inflammatory drugs (NSAIDs) are commonly used to control early inflammation by inhibiting the enzymes involved.^51^ Cox2 inhibitors and glucocorticoids have also been reported as additional options.^8^, ^52^ Traditional NSAIDs such as aspirin, indomethacin and ibuprofen have limited use due to their toxic effects on the gastrointestinal tract and kidneys.^53^, ^54^, ^55^ Radiation therapy can help inhibit granulation and osteogenic differentiation in MSCs, making it a viable option for the prevention or treatment of HO.^56^ The combination of NSAIDs and radiotherapy is currently considered the standard therapy for preventing the development of HO.^51^ Bisphosphonates have also been reported to delay the mineralization of HO by inhibiting the bone remodelling process, but there is insufficient evidence to support their effectiveness.^57^ For patients with preexisting HO formation, surgical resection measures, such as total hip arthroplasty (THA), are primarily employed.^58^ Although surgical resection improves the limitation of movement caused by heterotopic bone, it presents challenges due to the complex vascular composition of HO‐forming tissue, making complete removal difficult. Additionally, surgery may result in secondary injury from healed wounds and possible recurrence.^59^, ^60^ Postoperatively, NSAIDs are used to control inflammation, but they are less effective on their own in patients with advanced stages of HO progression.^61^, ^62^ Current researches focuse on therapies targeting the osteogenic process, including the development of drugs that target the BMP‐smad pathway.^7^, ^51^ However, therapeutic studies targeting the Wnt signalling pathway, which is widely implicated in the ossification process, are relatively lacking in the field of HO treatment.

### 4.1 RARγ agonists

**FIGURE 6:**
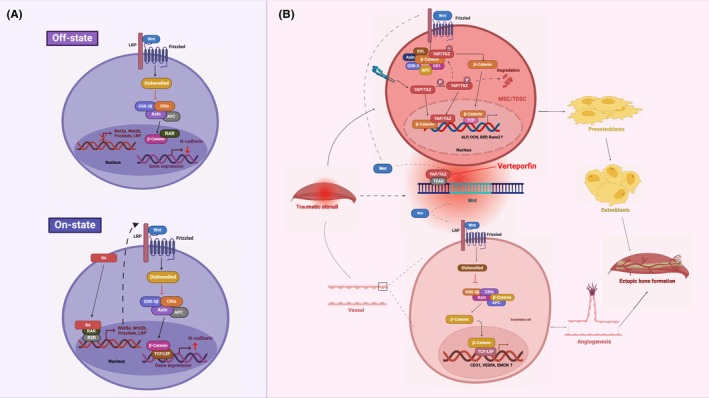
Mechanisms of RA and verteporfin in treating HO. (A) RA binds its intranuclear receptor RAR to promote the transcription of Wnt signalling components, including Wnt5a, Wnt2b, Fzd and LRP. Studies have shown that Wnt signalling could upregulate the expression of N‐cadherin to block chondrogenesis. (B) YAP‐β‐catenin is involved in angiogenesis and osteogenesis by regulating the expression of VEGFA, CD31, EMCN, ALP, RUNX2 and OCN. (Created with BioRender.com).

Recently, investigations have been conducted on drugs belonging to the retinoic acid class that have the potential to inhibit chondrogenesis. Retinoic acid (RA), also known as active vitamin A, is a potent inhibitor of chondrogenesis and exerts its effects by activating its receptor RAR. There are three forms of RAR receptors, namely, RARα, RARβ and RARγ.^63^ Isotretinoin, a full RAR pan agonist, has been found to prevent the onset of ossification in fibrodysplasia ossificans progressiva (FOP). However, its use is limited due to side effects such as skin problems and hair loss. Studies have demonstrated that selective activation of the RARγ receptor can inhibit the process of chondrogenesis in mesenchymal cells in vitro, while activation of RARα and RARβ does not have this effect.^63^ Consistently, Shimono et al. reported that RARγ selective agonists were more effective in blocking HO. They showed that oral RARγ agonists prevented the formation of HO and FOP with fewer side effects compared to RA application, potentially due to their specific activation. Parovalotene, a selective RARγ agonist, is currently being used to treat FOP.^7^ Mechanistically, RA inhibits the Smad pathway and enhances the Wnt signalling pathway. By targeting RARγ on mesenchymal stem cells (MSCs), RA reduces the phosphorylation of smad1/5/8, attenuates the signalling activity of the BMP pathway, and inhibits the onset of osteogenesis.^7^ Regarding its regulation of Wnt signalling, RARγ interacts with β‐catenin in the normal resting state. When it binds to agonists, β‐catenin is released and exerts transcriptional effects to inhibit chondrogenic differentiation.^64^ Therefore, RA or activation of RARγ can inhibit HO formation by strengthening the inhibitory effect of Wnt signalling on chondrogenic differentiation in MSCs ADDIN EN.CITE.^64^ (Figure 6).

### 4.2 Wnt inhibitors

Dkk‐1, SOST and SFRP‐1 are extracellular inhibitors of the Wnt signalling pathway. The first two inhibit Wnt signalling by binding to LRP, while SFRP‐1 acts as a decoy receptor to bind to Wnt ligands and inhibit their action.^65^, ^66^, ^67^ Niu et al. analysed the expression of Wnt inhibitors and bone turnover markers in the sera of patients with AS, diffuse idiopathic skeletal hyperostosis (DISH), OPLL and ossification of the yellow ligament (OYL) and found that OSC and Dkk‐1 were associated with excessive bone formation. On the one hand, the expression levels of OSC were high in the sera of all four patient samples, indicating ectopic bone formation activity.^68^ On the other hand, Dkk‐1 was low, suggesting hyperactivation of the Wnt pathway. Although SFRP‐1 serum levels were significantly elevated in DISH patients and SOST expression was elevated in OPLL compared to DISH patients, these changes could be counteracted by a decrease in Dkk‐1. These results suggest that changes in Wnt inhibitor expression may play a role in aberrant Wnt signalling.^68^ Similarly, Dong et al. found that serum levels of Dkk‐1 were lower in OPLL patients than in non‐OPLL patients, indicating that Dkk‐1 negatively regulates the development of OPLL.^69^ This suggests that understanding the development of Dkk‐1 and other molecular inhibitors may provide insights into the treatment of OPLL.^69^ Additionally, an inhibitory effect of transferrin receptor 2 (Trf2) on bone formation has been reported, affecting bone mass and ossification development through the regulation of BMP and Wnt signalling.^70^ In vivo and in vitro studies have shown that Trf2 interacts with BMP and its receptor to activate p38MAPK signalling, which induces the expression of SOST and Dkk‐1, thereby inhibiting Wnt signalling. Tfr2‐deficient osteoblasts express less Dkk‐1 and SOST, leading to increased bone mass and calcification, while overexpression of SOST restores normal levels of bone formation.^70^ Thus, Tfr2 can block heterotopic ossification induced by BMP and has the potential to be developed as a novel strategy for heterotopic ossification prevention (Table 2).

### 4.3 Verteporfin

Verteporfin (VP) is an antiangiogenic drug commonly used in clinical practice to inhibit retinal angiogenesis. When exposed to laser irradiation, VP induces apoptosis of endothelial cells by generating reactive oxygen species, thereby inhibiting angiogenesis. Additionally, VP acts as an inhibitor of the YAP pathway, preventing the binding of YAP to its transcription factor TEAD, which leads to YAP degradation.^22^ YAP is known to play a crucial role in angiogenesis and bone formation and is often coregulated with factors such as Runx2, smad and β‐catenin to promote osteogenic differentiation.^71^, ^72^ In a recent study, Guo et al. investigated the effects of VP on HUVECs and TDSCs through in vivo and in vitro experiments, respectively. They discovered that VP inhibits HO progression by suppressing angiogenesis and osteogenic activity. It was observed in vitro that treatment of VP downregulated the expression of the angiogenesis‐promoting molecules including VEGFA, CD31 and EMCN in HUVECs, and also downregulated the expression of the osteogenic markers OCN, Runx2, OPN and BSP in TDSCs extracted from trauma mouse model.^22^ Similarly, in vivo experiments confirmed the inhibitory effect of VP on the angiogenic capacity of HUVECs and the osteogenic potential of TDSCs, as well as on HO generation. More detailed, the expression of β‐catenin and YAP was significantly reduced and positively correlated in all experimental groups treated with VP.^22^ Therefore, Guo et al. concluded that VP inhibits angiogenesis and osteogenic differentiation through the YAP/β‐catenin axis. It was further supplemented by the fact that treatment of LiCl, an agonist of the Wnt signalling pathway, rescued the inhibitory effect of VP on angiogenesis and bone formation by restoring the expression of VEGFA, CD31, EMCN, ALP, RUNX2 and OCN.^22^ Overall, VP effectively attenuated H‐type angiogenesis and osteogenesis in the early stages of HO and showed potential for broadly attenuating HO formation in established cases. However, further clinical studies are needed to evaluate its effectiveness and safety (Figure 6) (Table 2).

## 5 DISCUSSION

Heterotopic ossification is a pathological process in which bone forms in soft tissues, involving the reactivation of signals from various physiological bone formation processes. Multiple studies have demonstrated the involvement of canonical Wnt signalling in typical HO and other ossification‐related diseases, including AS and ossification of the posterior longitudinal ligament OPLL, which plays a crucial role at different stages.^16^ Currently, the role of Wnt signalling in HO is mainly reflected in the promotion of chondrocyte hypertrophy and mineralization, as well as osteoblast differentiation. Recently, a research has suggested that Wnt signalling is involved in the angiogenesis of HO, promoting the generation of H‐type vessels and subsequent osteogenesis through the YAP/β‐catenin axis. Nonetheless, studies on the role of Wnt signalling in the early inflammatory process of HO are still lacking ADDIN EN.CITE, although the involvement of Wnt in other diseases, including cancers, has been implicated.

Many upstream factors contribute to aberrant activation or inhibition of Wnt signalling, including other signalling pathways and some osteogenic factors. BMP signalling, as one of the most profound signalling pathways involved in HO development, have been found to closely interact with Wnt signalling at different stages.^16^, ^39^ Their combinational expression has been observed in cases of rare HO in cancer. The relationship of HH and Wnt signalling has not been clearly implicated in the HO context, but β‐catenin seems to be necessary downstream for hh to induce HO.^42^ Recently, YAP was found to regulate Wnt signalling by either incorporating in the complex restricting β‐catenin or upregulating the expression of Wnt ligands.^22^ The relationship between YAP and β‐catenin might be worth further exploration since their cooperation has been implied in both angiogenesis and osteogenic activity. Additionally, mechanical stimuli, which have been reported to be responsible for some OPLL cases, also stimulate the YAP/β axis. In recent years, several studies have reported the involvement of various miRNAs. Among them, miR‐22‐3p derived from M2 inflammatory cells has been found to be aberrantly upregulated, weakening the inhibitory effect of PER2 on Wnt and triggering the ossification process in AS.^49^ It may suggests a regulatory role of Wnt signalling byproducts of inflammatory processes, which may be crucial for inducing ectopically expressed Wnt signalling. Other miRNAs, including miR‐487b, miR‐124, miR‐103 and other molecules, also play a significant role in the aberrant activation of Wnt signalling.^30^

Although Wnt signalling is involved in various ossification‐related diseases, its role varies depending on the specific disease context and the regulators involved. In the case of OPLL, Wnt inhibits chondrogenic differentiation and endochondral ossification of early MSCs,^34^ thereby hindering HO generation ADDIN EN.CITE.^34^ However, fibroblasts from AS patients showed reduced levels of Dkk‐1 and significantly elevated Wnt signalling which caused upregulation of c‐Myc and osteogenic markers (ALP, OCN and Runx2).^73^ In this context, Wnt/β‐catenin signalling promoted the proliferation and osteogenic activity of AS‐derived fibroblasts. These findings contradict the role of Wnt demonstrated in the previous study of OPLL. Additionally, a study on mechanical stress in OPLL showed the involvement of β‐catenin in causing ossification.^32^ Therefore, Wnt signalling may plays a crucial role in different stages of HO development and in the development of ossification in various disease contexts, and further research is needed to understand their mechanisms.

Currently, therapeutic and prophylactic measures for HO mainly focus on inflammation control, primarily through the use of nonsteroidal anti‐inflammatory drugs (NSAIDs). Surgical resection remains the standard approach to improving joint mobility in patients with formed bone tissue. However, these methods have significant limitations, including toxic effects, secondary trauma and recurrence. Moreover, their limited effectiveness poses challenges for treatment.^8^ Pharmacological treatments targeting cartilage and bone formation are still relatively inadequate, with investigations mainly directed at the BMP‐Smad pathway. Wnt inhibitors have been utilized in various cancers and some degenerative diseases, but there is still ample room for development in targeting therapeutic areas related to HO.^74^ A novel RARγ agonist was shown to be able to enhance Wnt signalling to block chondrogenesis. Recently, the effectiveness of verteporfin in inhibiting angiogenesis and subsequent osteogenesis through the YAP/β‐catenin axis has been shown through in vitro and in vivo experiments involving tendon‐derived stem cells (TDSCs) and human umbilical vein endothelial cells (HUVECs).^22^ Given the dynamic expression of Wnt signalling throughout HO development, inhibition or promotion of Wnt signalling alone may not be sufficient to prevent HO. Therefore, diagnostics used to identify the exact stage of patients might be of great significance so that drugs can be applied effectively.

In the future, further research is needed to elucidate the role of Wnt in early inflammatory processes and more trials in vivo are required to determine the effects of drugs. Additionally, the similarities and differences in the dynamic course of Wnt during ossification in different diseases and its interactions with other molecules remain largely unknown. Understanding these aspects may pave the way for future therapeutic approaches targeting canonical Wnt signalling.

## 6 SUMMARY

Wnt/β‐catenin signalling pathway has been shown to promote angiogenesis in the repair stage of HO through YAP/β‐catenin. Generally, the elevation of β‐catenin is linked to increased osteogenic differentiation, but it inhibits the development of ossification under the circumstance of OPLL. Additionally, canonical Wnt signalling promotes the chondrocyte maturation so that subsequent endochondral ossification can take place. Its interaction with other osteogenic factors and some miRNAs also regulates the process. In the future, therapeutical measures targeting Wnt β‐catenin signalling are in need of further exploration.

## AUTHOR CONTRIBUTIONS

**Yike Zhao:** Conceptualization (equal); investigation (equal); writing – original draft (equal); writing – review and editing (equal). **Fangzhou Liu:** Conceptualization (equal); investigation (equal); writing – original draft (equal); writing – review and editing (equal). **Yiran Pei:** Investigation (equal); writing – original draft (supporting); writing – review and editing (supporting). **Fengyu Lian:** Investigation (supporting); writing – original draft (supporting); writing – review and editing (supporting). **Hui Lin:** Conceptualization (equal); funding acquisition (lead); supervision (lead).

## FUNDING INFORMATION

This work was supported by the National Natural Science Foundation of China (32460216 to HL), and, Natural Science Foundation of Jiangxi Province of China (20224ACB206024, 20232BAB206081 and 20232BCJ23008 to HL).

## CONFLICT OF INTEREST STATEMENT

None of the authors have conflicts of interest to declare.

## Data Availability

The data that support the findings of this study are available from the corresponding author upon reasonable request.
